# Exploring Unprotected Anal Intercourse among Newly Diagnosed HIV Positive Men Who Have Sex with Men in China: An Ethnographic Study

**DOI:** 10.1371/journal.pone.0140555

**Published:** 2015-10-13

**Authors:** Haochu Li, Eleanor Holroyd, Joseph Lau

**Affiliations:** 1 School of Public Health, Shandong University, Jinan, China; 2 UNC Project-China, Institute for Global Health & Infectious Diseases, University of North Carolina at Chapel Hill, Chapel Hill, North Carolina, United States of America; 3 School of Health Sciences, RMIT University, Melbourne, Australia; 4 Centre for Women’s Health, Gender & Society, The University of Melbourne, Melbourne, Australia; 5 Jockey Club School of Public Health and Primary Care, The Chinese University of Hong Kong, Hong Kong, Hong Kong SAR; David Geffen School of Medicine at UCLA, UNITED STATES

## Abstract

**Background:**

Unprotected anal intercourse (UAI) is a major pathway towards secondary HIV transmission among men who have sex with men (MSM). We explored the socio-cultural environment and individual beliefs and experiences conducive to UAI in the context of Southern China.

**Methods:**

We employed an ethnographic approach utilizing a socio-ecological framework to conduct repeated in-depth interviews with thirty one newly diagnosed HIV positive MSM as well as participant observations in Shenzhen based healthcare settings, MSM venues and NGO offices.

**Results:**

Some men (6/31) reported continuing to practice UAI after an initial diagnosis of being HIV positive. For MSM who had existing lovers or stable partners, the fear of losing partners in a context of non-serostatus disclosure was testified to be a major concern. MSM with casual partners reported that anonymous sexual encounters and moral judgments played a significant role in their sexual risk behaviors. Simultaneously, self-reported negative emotional and psychological status, perception and idiosyncratic risk interpretation, as well as substance abuse informed the intrapersonal context for UAI.

**Conclusion:**

UAI among these HIV positive MSM was embedded in an intrapersonal context, related to partner type, shaped by anonymous sexual encounters, psychological status, and moral judgments. It is important that prevention and intervention for secondary HIV transmission among newly diagnosed HIV positive MSM in China take into account these contextual factors.

## Introduction

In China there are currently about 780,000 people living with HIV (PLWH). [1] The percentage of newly reported HIV cases attributed to male homosexual exposure has increased from 0.2% in 2001 [2] to 32.5% in 2009 [3] and HIV prevalence among Chinese men who have sex with men (MSM) reached 5.3%. [4] A high prevalence of unprotected anal intercourse (UAI) among HIV positive MSM has been reported, [5] while another study reported a reduction of sexual risk behaviors among HIV positive MSM as well as other at-risk populations. [6] Predictors of HIV transmission via HIV positive MSM include the use of stimulants and other drugs, low coping self-efficacy, failure to disclose one’s HIV serostatus to all partners, past sexual behaviors and intentionality of condom use. [7] It is further argued that the benefits of highly active antiretroviral therapy (HAART) and earlier diagnosis of the disease have been offset by an increase in risk behaviors. [8] Quantitative psychosocial assessments dominate the studies of sexual risk among HIV positive MSM, emphasizing the prevalence of risk behaviors and the statistical significance of associated factors, such as substance use [9] and a diagnosis of depression. [10] These studies however have underestimated the importance of understanding subjective experience, and therefore provided limited contextual and cultural insight on the occurrence of and meanings behind this behavior. Only a few qualitative studies have explored factors associated with UAI among HIV positive MSM, [11] combined with associated factors such as substance use, mental health, cultural norms and personal responsibility. [12]

There are limited studies to date that undertake a qualitative approach to explain UAI among HIV positive MSM within a Chinese context. In the reported study, a socio-ecological approach [13] was employed to enable a contextualization of the complex relationships and nuances of the interplay of UAI and related socio-ecological factors.

Prior to conducting the main study, the first author conducted a pilot study using a draft semi-structured interview guide in Mandarin Chinese with five HIV-infected MSM and five healthcare workers and volunteers independent from the main study, with modifications made accordingly. The socio-ecological approach, as supported by the pilot study, enabled an exploration of a broad range of personal and socio cultural factors within the context of contemporary Chinese society.

The current study conducted, in China, aimed to explore intrapersonal (e.g. individual characteristics of knowledge, attitudes, self-concept, emotion, behavior, and skills), interpersonal (e.g. significant others and interpersonal relationships), and socio-cultural (e.g. cultural context, social norms and networks) factors shaping the practice of UAI.

## Methods

### Settings and research design

In 2010 Shenzhen, China, had a population of 13.1 million people, of which over 80% of residents were internal migrants. [14, 15] In 2010, the number of MSM in Shenzhen was estimated to be approximately 100,000–200,000. [16] The first author conducted the formal ethnographic fieldwork in Shenzhen during January to September 2010. This research was undertaken in collaboration with a local grassroots HIV prevention organization (NGO) serving MSM with established connections to PLWH.

### Participants

The inclusion criteria for participation were: 18 years of age or older, diagnosed as HIV positive in the last six months (newly diagnosed), and identified as a MSM. We used purposive sampling based on diverse socioeconomic backgrounds to recruit participants. A total of 31 eligible newly diagnosed HIV positive MSM (Table 1) were interviewed, among which six participants (Table 2) reported UAI after being diagnosed as HIV positive.

**Table 1:** Summary of the characteristics of the newly diagnosed HIV positive MSM who took part in repeated in-depth interviews.

Characteristics	Repeated interviews	Total %(N = 31)
**Age**		
18–25	8	25.81
26–35	19	61.29
36–40	4	12.90
**Duration since diagnosis**		
1–2 months	15	48.39
3–4 months	11	35.45
5–6 months	5	16.13
**Occupations**		
Office	10	32.26
Service/seller	5	16.13
Technician	9	29.03
Labourer	4	12.90
Sex worker	1	3.23
Jobless	2	6.45
**Education**		
College	8	25.81
High/technological school	17	54.84
Secondary school	6	19.35
**Monthly income (CNY** ^**a**^ **)**		
More than 6,000	3	9.68
3,000–6,000	12	38.71
Less than 3,000	14	45.16
No income	2	6.45
**Sexual identity**		
Homosexual	28	90.32
Bisexual	3	9.68

^a^ One US Dollar was equal to 6.80 CNY in 2010.

**Table 2:** The characteristics of six participants who practiced unprotected anal intercourse after the diagnosis of HIV positive.

Pseudonym	Monthly income RMB(USD)	Education	Job	Age	Sexual identity	Registered permanent residence
Zhu	4000 (588)	High school	Office worker	27	homosexual	urban
Hei	1500 (221)	Secondary school	labour	25	homosexual	rural
Yan	4000 (588)	College	Office worker	30	homosexual	urban
Han	2000 (294)	High school	Office worker	25	homosexual	rural
Jin	3500 (515)	College	technician	32	homosexual	rural
Xie	2500 (368)	Vestibule school	labour	31	homosexual	rural

### Procedures

Ethics approval was granted by the Survey and Behavioral Research Ethics Committee in the Chinese University of Hong Kong. The collaborating NGO (the largest MSM volunteer workgroup in Shenzhen with about 50 active volunteers providing HIV/AIDS prevention and intervention services to MSM) approached prospective participants through their established networks and referred to us those who wanted to voluntarily participate. Written informed consent was obtained, assuring confidentiality and use of pseudonyms for any reported data. Subsequent interviews were conducted for each participant three months after the initial interview. Transcribed verbatim data from the initial interview was read. Incomplete saturation or missing points were explored in the second interviews. Each interview, both initial and subsequent, lasted between 1.5 to 2 hours and was conducted in a private room in the NGO. Participants were given a cash reimbursement (CNY 500 in total, approximately USD 80) to compensate for their time and transportation costs.

We used a life profile approach focusing on individual life narratives to provide background and context to our participants’ contemporary community lives. The first author also simultaneously conducted participant observations in MSM venues, NGO offices, and local health care settings in Shenzhen. Twelve men from our main study accepted the first author’s participatory engagement in their daily life, such as visiting their homes or work places, and hanging out with them.

### Data analysis

The tape-recorded interviews were transcribed verbatim into Mandarin Chinese. We employed thematic content analysis [17] concurrently with data collection in order to capture emerging themes. The analysis involved preliminary reading of transcripts and field notes, and identifying recurring themes. All authors discussed areas of emerging thematic importance and agreed on a set of preliminary codes. The adapted socio-ecological framework partially informed our grouping of informative quotes relevant to the themes, such as emotionality, cognition and substance use (intrapersonal level), interaction with partners (interpersonal level), gay sauna culture and moral judgment (socio-cultural level). We further refined the thematic structures based on ethnographic observations and collective discussions.

## Results

The majority of participants reported practicing safer sex after an initial diagnosis of being HIV positive, while the minority of participants reported UAI that may put their sex partners at risk of acquiring HIV**.** Some participants reported initially using condoms or practicing oral sex and masturbation, but then resorting to UAI again. We illustrate the practice of UAI underpinned by the socio-ecological factors as follows.

### Intrapersonal level

#### Emotional and psychological factors

Some participants reported experiencing negative emotions, such as moodiness, anger, and hopelessness after being diagnosed as HIV positive. Their emotions were seen to be related to uncertainty about sources of transmission, feelings of injustice and imbalance, psychological suffering and negative life attitudes, and feeling depressed or anxious. These emotional and psychological situations informed the intrapersonal context of practicing UAI. Hei expressed:

Shortly after being diagnosed as HIV positive… I became very “di luo” (moody and despairing). …After the diagnosis, I was really bad physically and always had insomnia, and therefore had thoughts death. …Now I don’t want to attend any activities organized by the uninfected. Even though others don’t know that I am an HIV-infected person, I have some thinking in my mind. I see others so happy, and then I will be in low spirits and feel stressed.

After becoming HIV infected, some participants felt that they now had nothing to lose or that nothing worse could occur in their lives. They didn’t think other diseases would matter and attributed this attitude to feelings of hopelessness and depression. For example, Han said:

The suffering this ‘shen fen’ (identity/status of being HIV positive) brings with me is that I feel ‘sheng bu ru si’ (a fate totally worse than death). …I feel that in the whole process of our life, the most serious issue is that once infected (with HIV) I cannot be cured. Other issues don’t matter, except AIDS.

Some participants reported initially using condoms or practicing oral sex and masturbation, but then resorting to UAI again. Feelings of being stressed, alone, and empty were expressed. As Xie said,

In T Sauna, someone sucked my dick. I wanted to reject him, but suddenly I felt quite ‘shuang’ (love freedom/very comfortable). I then didn’t reject him. …an Internet friend went to my home, but we didn’t do (anal sex). We performed mutual touching and masturbation.

In the follow-up interview, Xie testified that in the past three months, he practiced UAI on a monthly basis. He described,

In July, in T Sauna, an acquaintance met up with me and pulled me into a room. He used lubricant, but no condom [in the penetration]. … In August, in K Sauna, at the beginning we used a condom. But later he said it was uncomfortable and he then took the condom off. In September, one man wanted to penetrate me. Because there was no condom in the room (a sauna room). …We then didn’t use a condom to have sex.

Xie further explained,

I feel very depressed, very sad for my families, very stressful. …without boyfriends, I am alone, and empty. Sometimes, I had sexual desire, I therefore went to those places (gay saunas) [to have sex].

#### Perception and risk interpretation

After being diagnosed as HIV positive, some participants perceived that they had a low risk of HIV transmission dependent on who was “0” (the terminology for receptive anal sex commonly used among MSM in China) and who was “1” (the terminology for insertive anal sex commonly used among MSM in China). Jin’s situation provides such an example. A man penetrated Jin without using a condom and Jin claimed he performed “0”, as a strategy to avoid transmitting HIV to his partner. Jin said:

I dared not perform 1, because he is not HIV positive. If I perform 1, there is a high possibility of transmitting HIV to him. He is young. If I perform 1, I will hurt him, right. I can’t bear this guilt. I therefore performed 0.

Another perception existed that a diagnosis of HIV positive could be “zhuan yin” (changed to be negative) through taking HAART or Traditional Chinese Medicine (TCM). Zhu who practiced UAI with his boyfriend had a perception of the effects of “zhuan yin”, which could be argued to warrant public health service interventions since this could provide a rationale for continuing UAI. Zhu described:

Last month, I went back to be retested. My virus load dropped dramatically, and basically it was less than 500. General instruments cannot test it. That’s “zhuan yin”. It’s not positive, and it belongs to negative, that’s recessive. …it cannot spread out.

#### Substance use

Some participants mentioned substance use as a precursor to practicing UAI. Yan described how he had consumed alcohol when getting to know a boy in a sauna. One night they hung out drinking and then slept together. Yan said:

After drinking, I used no condom and penetrated him (the boy). …I was yun huhu (dizzy) and mimi huhu (dopey), and quite passionate.

### Interpersonal level

#### Non-disclosure of HIV status and partners’ refusal to use condoms

Some participants did not disclose their HIV positive status to their sex partners when refusing to use condoms. Some participants reminded their sexual partners to use a condom first, and if partners insisted on non-condom-use, they then testified that they did not feel responsible or guilty.

Zhu’s situation provides such an example. After becoming HIV positive, Zhu met a new boyfriend and started to develop a relationship with him. He did not dare to disclose his seropositive status and when they had sex, the man did not want to use a condom. When Zhu tried to negotiate condom use, the man asked: “Do you have diseases? Why bother?” Zhu was scared and said: “No, it is just for safety.” And then they had UAI.

#### Fear of losing partners

The fear of losing partners or the urge to develop a relationship reduced participants’ agency in serostatus disclosure and condom use. This diminished agency was embedded in a structural context of the reported difficultly of looking for lovers or stable partners within MSM circles. Jin’s situation provides such an example. A man approached him and he wanted to develop a relationship but this man did not like using condoms. In their first sexual encounter, Jin did not disclose his seropositive status in order to build up the relationship. Later, this man had anal sex with him twice without using a condom. Jin expressed:

I have come to Shenzhen for many years, but I don’t obtain my love. …at that time I was not strong enough to insist on using a condom, because he would suspect me (of being HIV positive). I was very conflicted in mind.

### Socio-cultural level

#### Culture of non-condom use in a tongzhi (gay) sauna setting

Some participants reported non-condom use as well as witnessing other men not using condoms in *tongzhi* saunas. The physical environment of a *tongzhi* sauna was seen as influential in condom use decision making in part due to the complexity and uncertainty, which was seen to attribute to a feeling of escaping social surveillance. In the observational fieldwork, some *tongzhi* saunas placed free condoms and lubricant on the counters. However these offers were not always taken up by the clients. The interaction in *tongzhi* saunas was observed to be orientated around the procurement of sex and seeking an instantaneous encounter. Some men reported to prefer unprotected instantaneous sex when they met potential partners because they felt that they may lose out if these partners went elsewhere or were approached by other men. In this specific circumstance, non-condom-use was normalized to some degree. Xie said:

In saunas, sometimes safe measures were not well practiced. Sometimes at midnight, someone sucked my dick, with no condom. In the steam room, someone sucked my dick, with no condom use…someone pulled me to penetrate him, using no condom. …Someone was penetrated by several men.

Some participants testified to having sex mainly in *tongzhi* saunas as a means of forgetting their seropositive status temporarily. After being diagnosed as HIV positive, Han commenced having sex solely in saunas. Han said: “They do not intentionally protect themselves. That’s a lot! …several men have sex together, without using a condom at all.” In the three-month interval between the two interviews, Han reported that he had visited saunas three times and had sex with up to six men and about half of whom with which he did not use condoms. Han said:

I don’t care whether they use condoms. …. It’s very dark in sauna rooms. No one knows about each other. It’s just for sex. After having fun, they will go home and cannot identify their partners. …I may forget my identity (HIV positive), and forget my stress.

#### Comparative moral judgment—“they deserve to be punished”

Some of the participants had specific moral attitudes towards specific sexual behaviors. These included having multiple sex partners, swallowing semen and having oral sex or group sex. Having HIV was regarded by some participants as a form of punishment. Some participants compared their behaviors with that of their partners as a rationale for practicing UAI. For example, Kong said, “I practiced some bad behaviors and therefore I got HIV infected; when you (partners) conduct such behaviors, you deserve to get it as well”. Another participant attributed his sexual partner’s “careless attitude” in not protecting himself to contributing to his practice of UAI. As Hei described:

He (a sex partner) said he has no feeling if using condoms so he doesn’t use condoms. When I had sex with him in 2008, he swallowed semen. …Anyhow, I have been infected. If he has not been infected, he will get infected sooner or later. …I hate to see those who are sexually promiscuous, swallow semen, like playing 69 (mutual oral sex) and “qun pi” (group sex). …I definitely use no condoms. I will definitely transmit (HIV) to them deliberately and let them realize that you may be lucky one time but you will not the second time.

## Discussion

In this study, an adapted socio-ecological model was used to underline and explore UAI among newly diagnosed HIV positive MSM in China enabling a multi-level and multi-dimensional analysis. The findings highlight intrapersonal contexts, partner type, the ensuing complexity between anonymous sexual encounters and psychological status, and moral judgments.

Our findings show that the intrapersonal context, such as emotionality, mental health, risk perception, and substance use, is an important domain related to UAI, which is consistent with previous studies. [18, 19] In the current study, some participants initiated safer sex after being newly diagnosed as HIV positive but subsequently reengaged in the practice of UAI when they felt under stress. Men with self-reported poor moods and holding a negative view of themselves or their futures (e.g., nothing to lose after becoming HIV positive) may well have a higher propensity to engage in unsafe sexual practices. This practice could result in increasingly negative views about popularized social models regarding condom use. [20]

Moreover, perceptions of self-rationalized optimism (e.g., “zhuan yin”) also warrants further attention with Chinese MSM populations. The perception of low HIV transmission risk from “0” to “1” accounted for the practice of UAI among some participants. Such findings are consistent with studies in the US, reporting that HAART reduced perception of negative health consequences (e.g., HIV transmission) and provided an added rationale for unsafe sex. [19, 21] Compared with men who reported no engagement with UAI, strategic positioning (serosorting) was associated with an increased risk of sexually transmitted infections, such as urethral gonorrhea and chlamydia. [22] MSM who had UAI with casual partners as well as MSM in serodiscordant relationships could be viewed as independent predictors of HIV seroconversion in Australia. [23]

Our study found that different partner typologies were differentially linked to the practices of UAI. For those HIV positive MSM with lovers or stable partners, fear of losing partners in a context of non-serostatus disclosure provided an important explanation for practicing UAI. Such a rationale is likely embedded in a social and cultural context with these Chinese HIV positive MSM finding it considerably more difficult to look for lovers or stable partners within MSM circles. This may be because in contemporary China homosexuals are excluded from cultural legitimacy. [24] Homosexuality in China has long been regarded as sick, abnormal or perverted, interwoven by traditional values of family, marriage, gender roles, and contemporary ideology of reformation and neo-liberalism. [25] A diagnosis of HIV may have further exacerbated these men’s suffering and sense of inequality.

In Shenzhen, it has been reported that 52.6% of MSM are not in steady relationships. [26] After being diagnosed with HIV, the MSM interviewed testified to needing emotional and social support, which was commonly associated with the fear of losing their partners. Moreover, non-disclosure of HIV status to sexual partners often coincided with the establishment of a new relationship. Internationally, it has been reported that a high percentage of HIV positive MSM who practiced unprotected sex did not disclose their HIV status to prospective sex partners before having unprotected sex. [27] An ethnography in the US indicated that few newly diagnosed HIV positive MSM had a consistent pattern of HIV disclosure since it is related to multiple and often competing emotional, situational, and legal factors. [28]

The fear of losing partners was reported to be an important reason for the practices of UAI among lovers or stable partners. In contrast, anonymous sexual encounters (e.g., *tongzhi* sauna) and the practice of moral judgment (e.g., equity) appeared to be closely related to UAI among casual partners. Anonymous sexual encounters have been repeatedly reported to promote unsafe sex. [12] Our analysis further emphasize that, for some newly diagnosed HIV positive Chinese MSM, anonymous sexual encounters coupled with the self-perceived psychological status was related to intrapersonal emotions, risk perception, and substance use. Our findings are consistent with an ethnographic study contending that experiencing negative emotions served to prompt unsafe sex in saunas. [29] A similar contention is applicable in *tongzhi* saunas in China, where unprotected sex was regarded by some MSM as a norm in this specific sexual space and as a way to satisfy certain emotional needs. For our participants who reported regularly visiting saunas and practicing unprotected sex, sexual gratification was testified to be a motivator justified by a lack of partners, loneliness, emptiness, boredom, or other social and psychological concerns.

For casual partners, the moral judgments of others within the domain of collectivistic and utilitarian Chinese morality was seen to be influential in the decision to practice UAI. Chinese society has been reported to be relatively more likely to punish people who practiced undesirable behaviors and to offer duty-based justifications than do their counterparts in the US. [30] Therefore, protecting oneself against HIV may be publically regarded as a personal duty, and anyone who violates this duty could be seen as deviating from this norm of equity, an underlying basis for many Chinese moral and ethical social dilemmas. [31] Our participants referred to equity and fairness as rationales for UAI with sex partners, who they regarded as deserving punishment. This finding is consistent with the equity theory in that individuals will become distressed when they find themselves participating in inequitable relationships subsequently leading to attempts to eliminate their distress through restoring equity. [32] A study in the US argued that HIV positive men who believe that other persons have intentionally tried to infect them are at especially high risk of engaging in UAI, due to attempting to restore a sense of equity. [33]

The current study is subject to limitations. Firstly, the use of digital recorders may have prohibited participants from openly talking about their stories of unsafe sex. Secondly, the sample size was small and sampling was limited to social networks of local MSM/PLWH groups mainly confined to the author’s ethnographic field. Thirdly, this manuscript has focused on the socio-ecological understanding of UAI, which was an element of the larger study. However, most of the participants reported practicing safer sex. Despite these limitations, we would contend that the current study has highlighted a Chinese socio-ecological context that may be related to UAI among newly diagnosed HIV positive MSM in a partner-specific manner. We contend that UAI among these HIV positive MSM was embedded in the Chinese socio-cultural environment, personalized belief systems, and experiences in notions of comparative moral judgment, *tongzhi* saunas, psychological status, partner types and negative intrapersonal contexts. It is important that prevention and intervention strategies for secondary HIV transmission among newly diagnosed HIV positive MSM in China take into account these specifics.
